# Autofluorescence enhances intraoperative detection of parathyroid glands: a single-center comparative study

**DOI:** 10.3389/fendo.2026.1788568

**Published:** 2026-03-31

**Authors:** Jean-Baptiste Dubuis, Jeremy Meyer, Maria Mavromati, Sophie Leboulleux, Frédéric Triponez, Marco Stefano Demarchi

**Affiliations:** 1 Department of Thoracic and Endocrine Surgery, University Hospitals of Geneva, Geneva, Switzerland; 2 Department of General, Visceral, and Endocrine Surgery, Pitié-Salpêtrière Hospital, AP-HP, Sorbonne University, Paris, France; 3 Division of Digestive Surgery, University Hospitals of Geneva, Geneva, Switzerland; 4 Department of Endocrinology, Diabetology and Metabolism, University Hospitals of Geneva, Geneva, Switzerland; 5 Department of Thoracic and Endocrine Surgery, University Hospitals and Faculty of Medicine of Geneva, Geneva, Switzerland

**Keywords:** hypocalcemia, hypoparathyroidism, near-infrared autofluorescence, parathyroid preservation, thyroidectomy

## Abstract

**Introduction:**

Permanent hypoparathyroidism after total thyroidectomy remains a major complication, occurring in close to 10% of cases and increasing risks of cardiac, renal, and metabolic morbidity. Near-infrared autofluorescence (NIRAF) imaging has emerged as a promising adjunct to improve parathyroid gland (PG) identification and preservation. This study evaluates the efficacy of NIRAF in enhancing intraoperative PG detection compared to conventional visual inspection.

**Methods:**

This single-center retrospective cohort study included 349 patients undergoing total thyroidectomy. Outcomes were compared between a control group managed with visual PG identification alone (n = 126, 2014–2016) and a NIRAF-assisted group using Fluobeam LX^®^ imaging (n = 223, 2021–2023). The primary outcome was the mean number of PGs identified intraoperatively. Secondary outcomes included postoperative calcium and parathyroid hormone (PTH) levels, rates of PG autotransplantation, and inadvertent PG removal.

**Results:**

NIRAF use was associated with significantly improved intraoperative PG identification, with a higher mean number of glands visualized compared to controls (2.91 ± 0.98 vs. 2.59 ± 1.02; p = 0.004). Postoperative PTH levels were significantly higher in the NIRAF group (3.64 ± 1.72 vs. 3.03 ± 1.40 pmol/L; p < 0.001). Although postoperative calcium levels were lower in the NIRAF group (2.21 ± 0.11 vs. 2.29 ± 0.12 mmol/L; p < 0.001), the incidence of severe hypocalcemia (≤2.0 mmol/L) was low and comparable between groups (3.1% vs. 1.6%; p = 0.497). Rates of postoperative PTH <1.1 pmol/L, PG autotransplantation, and reimplantation did not differ significantly. Inadvertent PG removal was more frequent in the NIRAF group (20.6% vs. 5.6%; p < 0.001).

**Discussion:**

NIRAF imaging improves intraoperative identification of PG and is associated with higher early postoperative PTH levels. Although hypoparathyroidism did not differ between the two groups, the effect of NIRAF on permanent hypoparathyroidism requires prospective evaluation, particularly when combined with perfusion assessment using indocyanine green (ICG) angiography. Standardized use and surgeon training are necessary to optimize clinical outcomes.

## 1 Introduction

Permanent hypoparathyroidism is a serious and feared complication of thyroid surgery, reported in approximately 10% of cases across multiple large databases (1–3). This condition not only increases the risk of cardiac and renal morbidities but also imposes a significant financial burden on healthcare systems (4). Preservation of parathyroid gland (PG) function following total thyroidectomy is crucial to prevent permanent hypoparathyroidism.

Historically, the detection and preservation of PGs relied on the surgeon’s ability to identify and safeguard these glands and their vascular pedicles with the naked eye. Close dissection to the thyroid gland was a fundamental principle of endocrine surgery to protect PG function. However, due to their small size and color similarity to fat tissue, even the most experienced surgeons may inadvertently excise or injure PGs (5).

Several techniques have been developed to help in the detection and preservation of PG. One promising method involves fluorescence imaging using near-infrared light (NIR). NIR helps to detect autofluorescence arising from the PGs, making intraoperative near-infrared autofluorescence (NIRAF) a valuable tool for locating these glands. When PGs are stimulated with near-infrared light at 785 nm, their natural fluorophore whose identity remains unknown emits light within the near-infrared range (700–900 nm) (6). This emitted light can be captured by camera systems and probe based system, providing real-time surgical guidance through imaging. Numerous studies have highlighted the efficacy of NIRAF in this regard (7).

Our study aims to explore the accuracy of NIRAF systems in detecting PGs compared to the naked eye alone.

## 2 Material and methods

**Figure 1:**
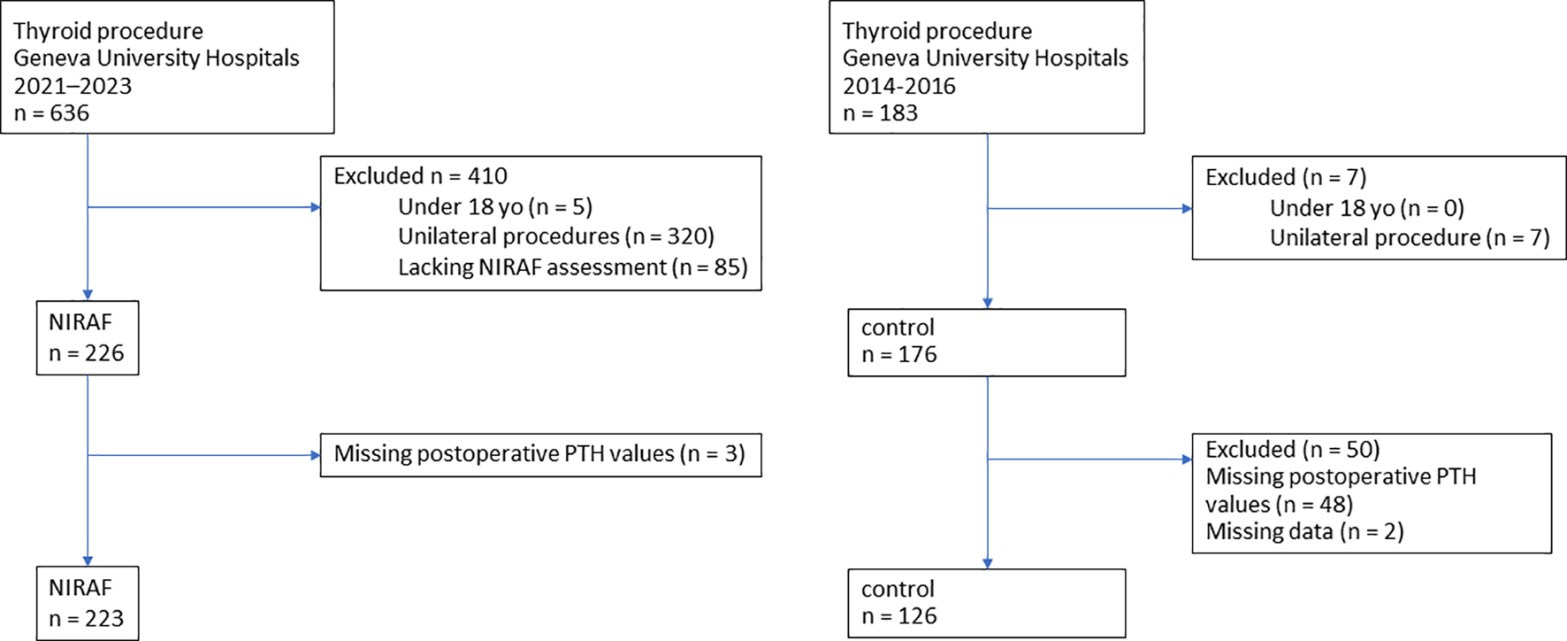
Study flowchart, NIRAF, Near-infrared autofluorescence; PTH, parathyroid hormone.

This single-center retrospective cohort study compared two groups of patients undergoing total thyroidectomy for various indications at the University Hospitals of Geneva. The historical control group consisted of patients operated on between September 2014 and February 2016, before near-infrared autofluorescence (NIRAF) technology was available. The data were already published by Vidal Fortuny et al. (8). The NIRAF group included patients operated between April 2021 and April 2023, during which an image-based NIRAF system was liberally used to assist PG visualization (**Figure 1**).

All surgical procedures were performed by an experienced endocrine surgeon following standardized protocols for total thyroidectomy.

In the NIRAF group, intraoperative identification of PGs was achieved using a Fluobeam LX^®^ device (Fluoptics, Grenoble, France), which detects near-infrared autofluorescence. ICG angiography was not employed in this group. Following thyroidectomy, the resected specimen was systematically examined ex vivo using the same NIRAF device to identify any parathyroid tissue.

In the control group, total thyroidectomy was performed without the use of NIRAF technology. PG identification relied exclusively on systematic visual inspection, with intentional exploration aimed at identifying all four glands after thyroidectomy. For each identified gland, intraoperative ICG angiography was performed using a near-infrared camera (Pinpoint^®^; Novadaq, Toronto, Ontario, Canada) to assess vascularization. The degree of fluorescence was graded according to a validated scoring system (9): ICG score 0 (black, indicating a non-vascularized gland); ICG score 1 (grey or heterogeneous, suggesting partial vascularization); or ICG score 2 (white, indicating a well-vascularized gland). In this group, the thyroid specimen was not routinely examined for parathyroid tissue after excision.

In both groups, the number of PGs visualized during surgery was prospectively recorded, along with the indications for and incidence of autotransplantation. Demographic characteristics, medical history, intraoperative findings, and postoperative biochemical results were collected retrospectively from electronic medical records.

Indications for autotransplantation were similar between groups. In the control group, PGs were autotransplanted when they could not be preserved *in situ*, when they were identified on the resected thyroid specimen, or when ICG angiography demonstrated absent perfusion (ICG score 0). In the NIRAF group, a more liberal approach to autotransplantation was adopted, which included glands that appeared ischemic on visual assessment (grey discoloration), could not be functionally preserved *in situ* due to close proximity to the thyroid capsule, or were identified on the ex vivo thyroid specimen.

All control patients received postoperatively 1 g of oral calcium and 800 IU of 25-hydroxyvitamin D twice daily, whereas NIRAF patients did not received any supplementation. The Calcium supplementation occurs after blood sample collection for Calcium and PTH.

The primary outcome compared PG identification between groups under conditions excluding indocyanine green (ICG) angiography. In the control group, identification relied exclusively on systematic visual exploration with active searching for all four PGs. In the intervention group, PGs were identified using NIRAF technology. All patients had standard follow‐up consisting of measurement of calcium and PTH levels on postoperative day 1. Secondary outcomes included postoperative serum calcium levels, analyzed as continuous variables and categorized according to the thresholds ≤2.2 mmol/L and ≤2.0 mmol/L, as well as postoperative PTH levels, reimplanted PGs and inadvertent PG removal. Inadvertently resected PG was defined as parathyroid tissue reported on the thyroid gland specimen in the pathology report. Multivariable linear regression models were constructed to assess the independent association between NIRAF use and the number of intraoperatively inspected PGs (primary outcome), postoperative serum calcium levels and PTH levels. All models were adjusted for clinically relevant covariates: Graves’ disease, malignancy, central neck dissection, age, and sex.

The Central Clinical Laboratory of the University Hospitals of Geneva performed all blood analyses. Calcium levels were adjusted according to serum albumin (calciumcorrected = (40−albumin(g/l))/200 + calcium_measured_). The normal range at the authors’ institution was 1.1–6.8 pmol/l for PTH and 2.20–2.52 mmol/l for calcium. Low PTH was defined as PTH < 1.1 pmol/l. Hypocalcemia was defined as corrected calcium level ≤ 2.20 mmol/l. Severe hypocalcaemia was defined by a corrected calcium level ≤ 2.00 mmol/l and hypoparathyroidism by a PTH level < 1.1 pmol/l. Permanent hypoparathyroidism was defined as a PTH level <1.1 pmol/L persisting at 6 months postoperative.

Statistical analyses were performed using R (version 4.5.1; R Foundation for Statistical Computing, Vienna, Austria). Continuous variables were expressed as mean ± standard deviation and compared using Student’s t-test or Wilcoxon rank-sum test according to distribution normality and variance homogeneity. Categorical variables were expressed as counts and percentages and compared using chi-square test, or Fisher’s exact test as appropriate. In multivariable linear regression models Adjusted beta coefficients (β) with 95% confidence intervals (CI) and p-values were reported. The collinearity was assessed using variance inflation factors (VIF), with VIF < 2 indicating no concerning collinearity. A two-sided p-value less than 0.05 was considered statistically significant.

## 3 Results

A total of 819 patients were initially identified during the study period. From the 636 patients in the NIRAF group, patients under 18 years of age (n = 5) and those not meeting the inclusion criteria—defined as having undergone unilateral procedures (n = 320) or without NIRAF assessment of PGs (n = 85) were excluded, leaving 226 eligible NIRAF cases. Among these, patients with missing postoperative PTH values (n = 3) were further excluded. The final NIRAF group therefore comprised 223 patients (**Figure 1**).

183 patients were initially identified as control. Patients under 18 years of age (n = 0) and those not meeting the inclusion criteria—defined as having undergone unilateral procedures (n = 7)—were excluded, resulting in 176 control cases. Patients with missing postoperative PTH measurements (n = 50) were then excluded, yielding a final total of 126 eligible control cases (**Figure 1**).

Baseline demographic characteristics were comparable between groups. The proportion of female patients was similar (79.4% in the control group vs. 79.4% in the NIRAF group, p = 1.000). The mean age did not differ significantly between groups (51.00 ± 14.79 years vs. 50.91 ± 15.439 years, p = 0.959) (**Table 1**).

**Table 1:** Baseline characteristics of patients by NIRAF status.

	Control	NIRAF	p-value
	126(100)	223 (100)	
Preoperative variables			
Female, n (%)	100 (79.4)	177 (79.4)	1.000^1^
Male, n (%)	26 (20.6)	46 (20.6)	
Age, mean (SD)	51.00 (14.79)	50.91 (15.39)	0.959^2^
Diagnosis			
Graves disease, n (%)	24 (19)	65 (29.1)	<0.001^1^
Multinodular goiter, n (%)	63 (50)	50 (22.4)	
Cancer, n (%)	33 (26.2)	71 (31.8)	
Toxic multinodular goiterm n (%)	6 (4.8)	31 (13.9)	
Other indicationm n (%)	0 (0.0)	6 (2.7)	
Operation			
Central lymphe node dissectionm n (%)	18 (14.3)	49 (22.0)	0.086^1^
Final diagnosis			
Benign condition, n (%)	109 (86.5)	147 (65.9)	0.001^1^
Malignant condition, n (%)	17 (13.5)	76 (34.1)	

NIRAF: Near Infrared Autofluorescence, Values are mean (SD) for continuous variables or n (%) for categorical variables. ^1^Chi-square test, ^2^student t-test.

Surgical indication differed significantly between groups (p < 0.001). Graves’ disease was more common in the NIRAF group compared with the control group (29.1% vs. 19.0%), whereas multinodular goiter was more prevalent in the control group (50.0% vs. 22.4%). Total Thyroidectomy for cancer was 26.2% in the control group and 31.8% in the NIRAF group. Toxic multinodular goiter occurred more frequently in the NIRAF group (13.9% vs. 4.8%). Other surgical indications were rare in both groups (**Table 1**).

There was a trend toward more frequent central lymph node dissection in the NIRAF group, although the difference between the two groups was not statistically significant (14.3% vs. 22%, p = 0.086) (**Table 1**).

Final histopathological analysis revealed a higher proportion of malignant conditions in the NIRAF group compared with the control group (34.1% vs. 13.5%, p = 0.001) (**Table 1**).

### 3.1 Primary outcome

The mean number intraoperatively of detected PGs was significantly higher in the NIRAF group compared with the control group (2.91 ± 0.98 PGs vs. 2.59 ± 1.02 PGs respectively, p = 0.004) (**Table 2**).

**Table 2:** Primary and secondary outcomes by NIRAF status.

Primary and secondary outcomes				
Primary outcome		Control (n=126)	NIRAF (n=223)	p-value
Inspected glands, mean (SD)		2.59 (1.02)	2.91 (0.98)	0.0041
Secondary outcomes				
Postoperative PTH, pmol/l, mean (SD)		3.03 (1.40)	3.64 (1.72)	<0.001^1^
Low PTH, n (%)		4 (3.2)	7 (3.1)	0.6612
Reimplanted glands, n (%)		39 (31)	87 (39)	0.0743
Postoperative calcium, mmol/l, mean (SD)		2.29 (0.12)	2.21 (0.11)	<0.001^1^
Postoperative calcium ≤ 2.20 mmol/l, n (%)		27 (21.4)	116 (52)^3^	<0.001^3^
Postoperative calcium ≤ 2.0 mmol/l, n (%)		2 (1.6)	7 (3.1)	0.4972
Inadvertent PG removal, n (%)		7 (5.6)	46 (20.6)	<0.001^3^

NIRAF: Near Infrared Autofluorescence, Values are mean (SD) for continuous variables or n (%) for categorical variables. ^1^student t-test, ^2^Fisher exact test, ^3^Chi-square test.

In multivariable analysis adjusting for Graves disease, malignancy, central neck dissection, age, and sex, NIRAF remained independently associated with a higher number of inspected glands (adjusted β = 0.27, 95% CI [0.04, 0.50], p = 0.020). Collinearity was low across covariates (VIF < 2) (**Table 3**).

**Table 3:** 1 Adjusted for age, sex, Graves’ disease, malignancy, and lymph node dissection, NIRAF, Near Infrared Autofluorescence; CI, confidence interval.

Multivariable Adjusted Analysis				
Effect of NIRAF on outcomes				
Outcome	Adjusted Beta^1^	CI 2.5%	CI 97.5%	p-value
Inspected glands	0.27	0.043	0.496	0.02
Postoperative calcium	-0.073	-0.098	-0.047	<0.001
Postoperative PTH	0.663	0.304	1.022	<0.001

^1^Beta Adjusted for age, sex, Graves’ disease, malignancy, and lymph node dissection.

### 3.2 Secondary outcomes

Postoperative PTH levels at day1 were significantly higher in the NIRAF group (3.64 pmol/l ± 1.72 versus 3.03 ± 1.40, p < 0.001). No statistical difference in the low PTH level (PTH level < 1.1 pmol/l) was found between NIRAF (3.1%) and the control group (3.2%). No statistical difference in the proportion of patients with reimplanted PGs was found: 39% in the NIRAF group versus 31% in the control group (p = 0.075) (**Table 2**).

In multivariable analysis, NIRAF use was associated with higher PTH (adjusted β = 0.66 pmol/L, 95% CI [0.31, 1.02], p < 0.001) (**Table 3**).

Mean postoperative calcium concentrations were lower in the NIRAF group compared with the control group (2.21 ± 0.11 mmol/l vs. 2.29 ± 0.12 mmol/l, p < 0.001). In multivariable analysis, NIRAF use was associated with a decreased postoperative calcium concentration (adjusted β = -0.073 mmol/L, 95% CI [-0.098, -0.047], p < 0.001), after adjusting for covariates. (**Table 3**) Hypocalcemia (calcium level ≤ 2.2 mmol/l) occurred more frequently in the NIRAF group (52% vs. 21.4%, p < 0.001). Severe hypocalcemia (calcium level ≤2.0 mmol/l), was infrequent and did not differ significantly between groups (3.1% vs. 1.6%, p = 0.497).

Of patients with low PTH (3.2% control vs 3.1% NIRAF), only one patient per group had persistent low PTH at postoperative day 10, and persistent hypoparathyroidism at 6 months was observed exclusively in one patient of the control group. Inadvertent PG removal was more frequent in the NIRAF group compared to the control group (20.6% vs. 5.6%; p < 0.001).

## 4 Discussion

In this study, NIRAF improved PG identification during total thyroidectomy, with a higher mean number of glands visualized compared with active search alone in the control group. These findings are consistent with the meta-analysis by Canali et al., which reported an increased number of PGs identified using NIRAF (standardized mean difference: 0.33) (10). Similarly, a recent randomized controlled trial by Cousart et al. demonstrated an 17.9% improvement in gland identification using probe-based NIRAF technology (11). In line with these results, Takahashi et al. (12) reported in a large meta-analysis that NIRAF significantly increased the number of PGs identified, reduced inadvertent resections, and improved overall PG preservation (12).

Mean postoperative PTH levels were significantly higher in the NIRAF group compared with the control group (3.64 ± 1.72 vs. 3.03 ± 1.40 pmol/l, p < 0.001). After multivariable adjustment for age, sex, Graves’ disease, malignancy, and lymph node dissection, NIRAF remained independently associated with higher PTH (adjusted β = 0.66 pmol/L, 95% CI [0.31, 1.02], p < 0.001) highlighting the benefit of this technology. This result may be protocol-related, as some control-group patients with good PG perfusion on ICG angiography did not have PTH measured on postoperative day 1 and were excluded of our analysis, potentially resulting in a lower mean PTH concentration in the control group by excluding normal values (8).

Mean postoperative calcium concentrations were lower in the NIRAF group compared to the control group (2.21 ± 0.11 mmol/L vs. 2.29 ± 0.12 mmol/L, p < 0.001). As calcium levels were measured on postoperative day 1, prior to the administration of routine supplementation with oral calcium and vitamin D, these values were intended to reflect endogenous parathyroid function rather than the effect of supplementation (8).

However, this supplementation protocol constitutes a major potential confounder: patients who developed symptoms of hypocalcemia (e.g., paresthesia) may have received supplementation before blood sample collection, and these cases were not systematically reported in our dataset. Consequently, the observed difference in calcium levels may not solely reflect improved parathyroid preservation associated with NIRAF, but also the confounding influence of systematic or symptom-triggered supplementation in the control group.

After multivariable adjustment for age, sex, Graves’ disease, malignancy, and lymph node dissection, NIRAF remained independently associated with lower calcium levels (adjusted β = -0.07; 95% CI, -0.10 to -0.05; p < 0.001).

Our findings are consistent with those of Huang et al., who reported significantly lower calcium levels with NIRAF (2.15 vs. 2.22 mmol/L, p = 0.017) (13). However, Wolf et al. found no difference between groups (2.05 ± 0.11 vs. 2.09 ± 0.14 mmol/L, p = 0.22) (14), possibly due to differences in supplementation protocols or surgical techniques. Therefore, the inconsistent standardized supplementation protocols across studies highlights the need for cautious interpretation of postoperative calcium as a surrogate marker of parathyroid function.

Baseline characteristics reflected a significant case-mix imbalance, with NIRAF used more often in complex procedures involving Graves’ disease (29.1% vs. 19.0%) or malignancy (31.8% vs. 26.2%, p=0.001). As these conditions are associated with higher technical demands and risk of parathyroid injury, this preferential use represents a significant confounder. To address this, we performed a multivariable analysis adjusting for age, sex, Graves’ disease, malignancy, and lymph node dissection. This analysis confirmed that NIRAF was an independent predictor of improved intraoperative and postoperative outcomes: it was associated with a higher number of PGs inspected (Adjusted β=0.27, p=0.020) and higher postoperative PTH (Adjusted β=0.66, p<0.001).

This context may also explain the findings on hypocalcemia. Hypocalcemia (≤2.2 mmol/l) occurred more frequently in the NIRAF group, while severe hypocalcemia (≤2.0 mmol/l) did not differ significantly between groups (3.1% vs. 1.6%, p = 0.497). These results contrast with Canali et al., who reported a reduced risk of postoperative hypocalcemia using NIRAF (RR 0.65, 95% CI 0.50–0.84), and with Takahashi et al., who confirmed in a large meta-analysis of 4,281 patients that NIRAF significantly reduces temporary hypocalcemia (OR 0.56, 95% CI 0.43–0.72, p < 0.001) and temporary hypoparathyroidism (OR 0.56, 95% CI 0.40–0.79, p < 0.001), but has no significant effect on permanent hypocalcemia or hypoparathyroidism (10, 12).

Notably, two meta-analyses (Canali et al. and Rao et al.) that included randomized controlled trials of patients undergoing surgery with NIRAF found a statistically significant reduction in the risk of postoperative hypocalcemia defined as calcium < 2.0 mmol/L. These findings support the use of NIRAF in thyroid surgery, particularly in higher-risk cases (10, 15).

Another consideration is that NIRAF-assisted procedures may require less extensive dissection around the PGs, potentially minimizing disruption of their vascular supply. However, reduced dissection alone does not necessarily translate into improved functional outcomes, and the relationship between surgical extent, dissection strategy, and postoperative PG function warrants further study.

Given the single case of permanent hypoparathyroidism in the cohort, the study was underpowered to evaluate long-term outcomes, and NIRAF’s effect on permanent hypoparathyroidism remains uncertain. The literature suggests that device type, surgeon experience, and center volume may affect outcomes, emphasizing the need for cautious interpretation and further validation (1).

Some authors mentioned that visualization of the PG alone may not be sufficient to prevent hypoparathyroidism without assessment of vascular integrity. This limitation highlights the potential value of integrating perfusion assessment into surgical protocols. Studies by Demarchi et al. and Rossi et al. have shown that combining NIRAF with ICG angiography enables both identification and vascular evaluation of PGs (5, 16). Finally, introduction of PG angiography may enhance functional preservation of PG as described by Demarchi et al. (17).

Parathyroid reimplantation was slightly more frequent in the NIRAF group (39% versus 31% in the control group p = 0.074), contrasting with the findings of Benmiloud et al. and Rossi et al., who reported fewer reimplantation with NIRAF use (2, 16). This difference may be explained by methodological aspects of our practice: specimens were assessed with NIRAF after excision, leading to higher detection of parathyroid tissue. Furthermore, we applied a relatively liberal policy of autotransplantation, reimplanting glands whenever vascularization appeared inadequate or when ischemic changes were suspected. In the control group, intraoperative ICG-angiography was performed to assess PG vascularization. Glands considered devascularized were subsequently autotransplanted. This approach may have influenced the observed rate of parathyroid autotransplantation in the control group compared to the NIRAF group.

Several authors, including Annebäck et al. and Rao et al., have identified heterogeneity in surgical protocols and follow-up duration as key limitations in the literature (1, 15). Our study shares many of these constraints, including restriction to total thyroidectomy cases without stratification by lymph node dissection, lack of documentation on the timing of NIRAF application relative to dissection, absence of reliable data on inadvertent PG removal in the control group and standardization on the pathological analysis. To address these limitations, future multicenter randomized controlled trials should standardize NIRAF application (pre- vs post-dissection), integrate ICG perfusion assessment to NIRAF, and adopt uniform diagnostic criteria for permanent hypoparathyroidism. As suggested by Canali et al., structured training programs for surgeons may also enhance the effectiveness of NIRAF, particularly in low-volume centers (10).

In conclusion, NIRAF significantly improves PG identification though its impact on severe postoperative hypocalcemia and low PTH remains uncertain. Moreover, NIRAF requires further validation in long-term studies and high-volume procedures for its effect of permanent hypoparathyroidism. Standardized protocols, surgeon training, and cost-effectiveness analyses will be essential for translating these technological advances into consistent, widespread clinical benefit.

## Data Availability

The raw data supporting the conclusions of this article will be made available by the authors, without undue reservation.
